# Tape suture constructs for instabilities of the pubic symphysis: is the idea of motion preservation a suitable treatment option? A cadaver study

**DOI:** 10.1007/s00402-022-04547-6

**Published:** 2022-07-13

**Authors:** Adrian Cavalcanti Kußmaul, Fanny Schwaabe, Manuel Kistler, Maximilian Jörgens, Korbinian F. Schreyer, Axel Greiner, Wolfgang Böcker, Christopher A. Becker

**Affiliations:** Department of Orthopaedics and Trauma Surgery, Musculoskeletal University Center Munich (MUM), University Hospital, LMU Munich, Munich, Germany

**Keywords:** Pelvic instability, Biomechanics, Minimally invasive, Flexible osteosynthesis, Tape suture, Pubic symphysis

## Abstract

**Introduction:**

Current gold standard for the treatment of symphyseal disruptions includes anterior plating, almost entirely prohibiting symphyseal mobility and resulting in an iatrogenic arthrodesis followed by high rates of implant failure. Minimally invasive tape suture constructs have been found to maintain the micro mobility of ligamentous injuries, yet still providing sufficient biomechanical stability. Recently, this technique has been primarily investigated for symphyseal disruptions on synthetic pelvic models. Therefore, the aim of this study was to examine the feasibility of this novel flexible osteosynthesis on cadaveric pelvic models based on the following hypothesis: tape suture constructs ensure sufficient biomechanical stability without inhibiting micro mobility of the pubic symphysis for the treatment of symphyseal disruptions and maintain stability during long-term loading.

**Materials and methods:**

9 cadaveric anterior pelvic rings were used in this study and a symphyseal disruption was created in every specimen. The specimens were then exposed to short- and long-term vertical and horizontal cyclic loading after treatment with a tape suture construct in criss-cross technique. The mean maximum displacement (mm) during cyclic loading and the corresponding stiffness (N/mm) were measured and compared.

**Results:**

Regarding both displacement (mm) and corresponding stiffness (N/mm), the tape sutures displayed a significant difference between short- and long-term loading for cranial and caudal vertical loading (*p* < 0.01) but differences remained non-significant for horizontal loading (*p* > 0.05). No tape suture suffered from implant failure during long-term loading.

**Conclusions:**

The tape suture construct displayed sufficient biomechanical stability without exceeding the physiological mobility of 2 mm of the pubic symphysis; however, also maintained the desired micro mobility of the affected joint necessary to prevent an iatrogenic arthrodesis. Further, all tape sutures maintained stability throughout long-term loading.

## Introduction

Open book fractures, classified as AO type 61-B2 or 61-B3 fractures, are commonly the result of high impact trauma [1]. Symphyseal disruption in these cases is most often based on an anteroposterior pelvic compression [2]. Approximately 22% of patients with injuries of the pelvic ring display concomitant symphyseal instability, predominantly in the setting of an AO type B or C injury [3].

Open reduction and internal fixation (ORIF) with symphyseal plating is the current standard surgical treatment, aiming to restore pelvic stability [3–8]. Yet, symphyseal plating is frequently not only associated with infection, extensive surgery, high blood loss, hematoma or heterotopic ossification, but also bears the risk of implant failure, which can be observed in up to 81% of the cases [5, 9]. From a biomechanical perspective, this failure results from the unnatural attempt of an iatrogenic arthrodesis of the pubic symphysis as an originally dynamic junction with a physiological movement of up to 2 mm [5, 10].

Consequently, several approaches have been made to address this issue, including wires, cable cerclages and endobuttons, stressing the need for new technologies, especially given the fact of a pending enforcement of such a promising technique [11, 12].

Tape suture techniques for the treatment of ligamentous injuries of weight-bearing joints represent another promising alternative that are furthermore already successfully implanted for ligamentous stabilization of the knee [13], shoulder [14] or ankle [15]. These minimally invasive anchor systems avoid an iatrogenic arthrodesis by permitting physiological micro mobility, yet—based on the reinforcement of ligamentous structures and the splinting of the associated injury—without compromising biomechanical stability [16, 17]. In detail, this provision of a multiplanar stability is based on their criss-cross configuration with two tapes spanning between four anchors, similar to a modified SpeedBridge™ (Arthrex^®^, Naples, FL, USA). Also, this technique not only reduces perioperative risks due to its minimally invasive implantation [5], but also could avoid revision surgery for elective implant removal or implant failure [16, 18, 19].

Recently, Kussmaul et al. evaluated such a tape suture technique for instabilities of the pubic symphysis in a synthetic pelvic model with promising biomechanical results [16]. Data investigating the biomechanical properties of these constructs in a cadaver model and the concomitant transfer of their in-vitro feasibility, however, are still lacking.

Therefore, this study aims to examine the biomechanical properties of this flexible osteosynthesis in cadaver models based on the following two questions:

1. Does the tape suture construct provide sufficient biomechanical stability for the treatment of a symphyseal disruptions without compromising the physiological mobility in a cadaver model?
2. Is the tape suture able to resist long-term loading without implant loosening or failure?

## Materials and methods

After obtaining approval from the ethics committee (No. 210-16) and written consent from the relatives of the donors, a total of 9 cadaveric anterior pelvic rings were used in this study. The mean age of the specimens was 65 years, ranging from 25 to 86 years. Average bone mineral density (BMD), measured at the lumbar vertebrae L3-L5, was 129.7 ± 28.2 mg Ca-Ha/ml.

**Fig. 1:**
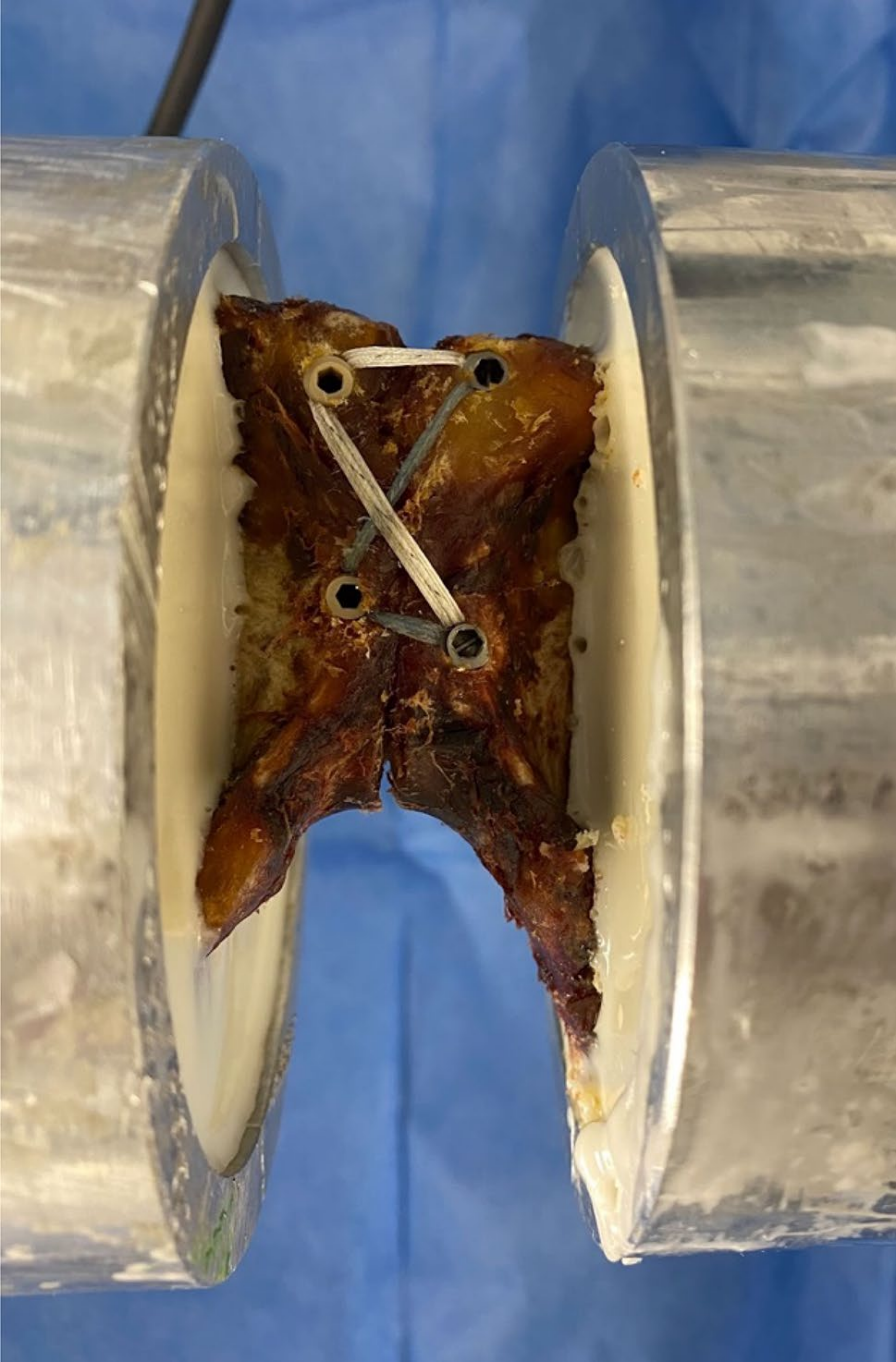
Applied criss-cross technique on completely transected and embedded symphysis

One day prior to the experiment, the specimens were taken out of the freezer to thaw. On the actual day of the experiment, the cadaver pelvises were heated in a water bath of 37 °C to approximate body temperature. Afterward, all symphyseal ligaments were dissected, simulating a symphyseal disruption. Next, the specimens were treated with a tape suture construct in criss-cross technique. In detail, according to Kußmaul et al. [16], the criss-cross technique was applied by first drilling two holes 2–3 cm below each other approximately 1 cm lateral to the symphyseal gap on each side. All holes were previously predrilled. Then, two PEEK SwiveLock anchors 4.75 mm (Arthrex^®^, Naples, FL, USA) loaded with one FiberTape^®^ each were placed on the right side of the pubic symphysis, after which the bands were spanned in a criss-cross manner and contralaterally fixed under appropriate tension with 6.5 mm titanium corkscrews^®^ (see Fig. 1).

**Fig. 2:**
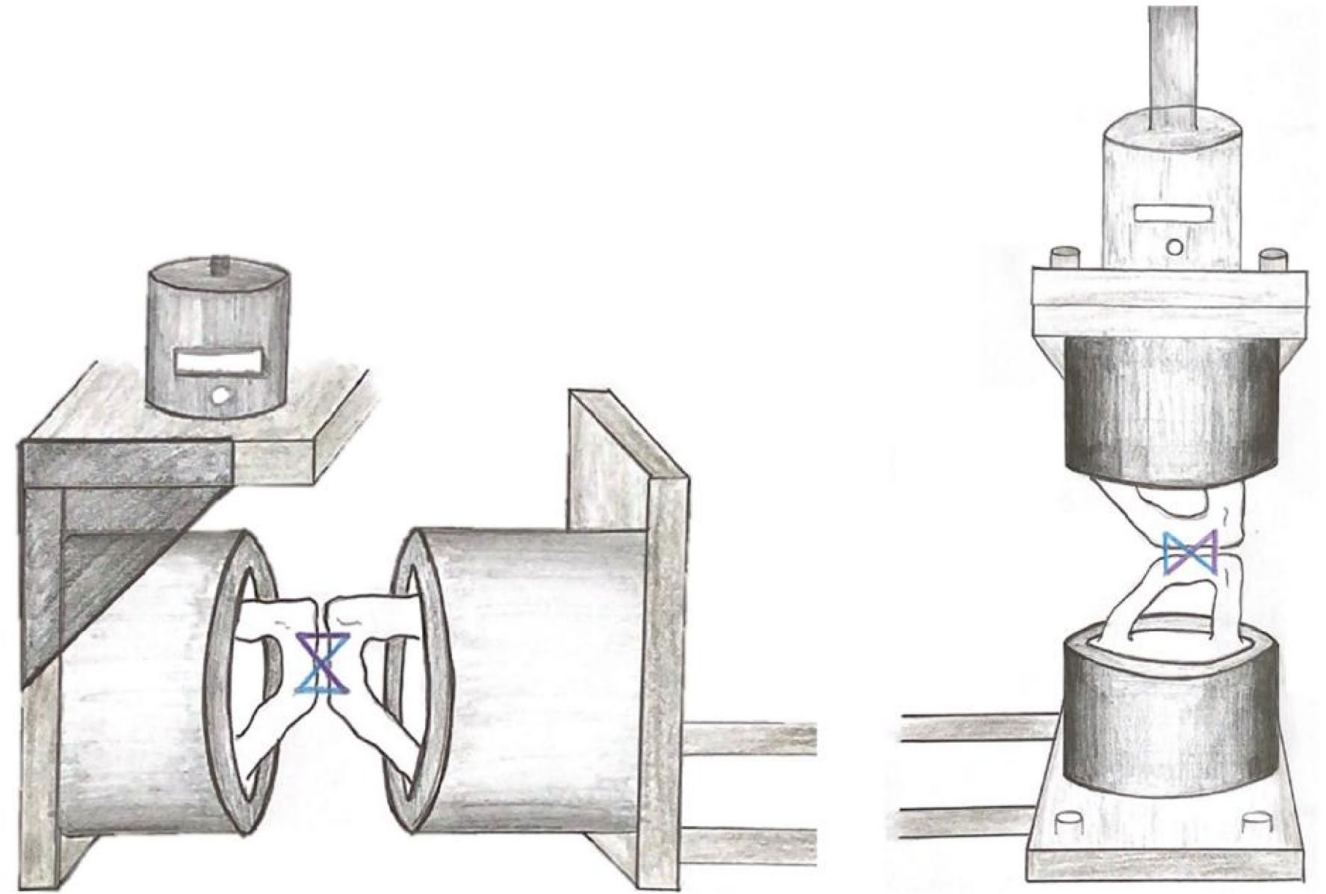
Biomechanical set-up with vertical loading on the left and horizontal loading on the right side

After symmetrical embedding of the isolated anterior pelvic rings in resin (RenCast^®^, Huntsman Cooperation^®^, Salt Lake City, UT, USA), the specimens were mounted on an all-electric industrial loading machine (Instron ElectroPuls™, E10000 Linear-Torsion, Norwood, MA, USA) (see Fig. 2).

**Fig. 3:**
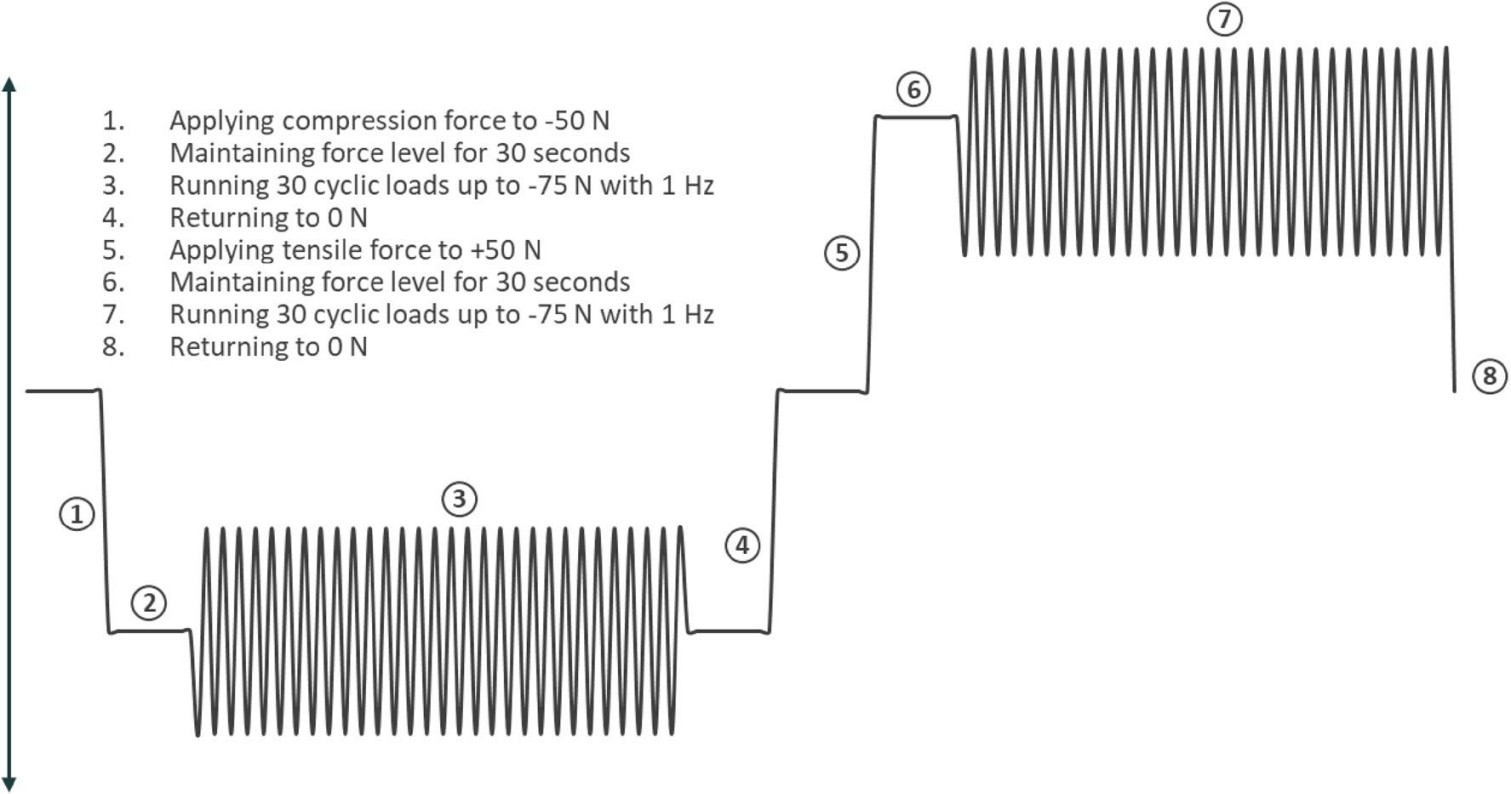
Testing protocol

For biomechanical testing, an 8-step testing protocol, equivalent to the previously mentioned reference study [16], was applied (see Fig. 3). Loading was applied horizontally first with 30 cycles of both tension and compression, followed by 30 cycles of vertical cranial and caudal loading to simulate short-term forces acting on the symphyseal joint while sitting, standing and walking [16] (see Figs. 2 and 3).

After completion of the 8-step protocol consisting of 30 cycles in each direction (see Figs. 2 and 3), the specimens underwent 1000 cycles of both vertical and horizontal loading with forces ranging from—75 N–+ 75 N (Δ 150 N) based on the previous protocol, resulting in a total of 4000 cycles and simulating long-term loading to ultimately examine the frequency of occurrence of implant loosening or failure.

During loading, symphyseal displacement (mm) and corresponding force (N) were measured at all times. For statistical analysis, maximum mean displacement (mm) as well as stiffness (N/mm) was calculated. Considering statistical analysis, the last 30 out of the 1000 long-term cycles were taken into account and compared to the initial values for each direction.

Data were statistically analyzed using IBM SPSS Statistics (Windows, Version 26.0, IMB Corp., Armonk, N.Y., USA). Based on literature [20], a previously performed case number estimation resulted in a required sample size of 9 with a power of 94% and a significance level of *p* < 0.05. Whenever the values followed normal distribution, a paired t-test was used for comparison. Otherwise, a Wilcoxon test was performed. To ensure normal distribution, the Kolmogorov–Smirnov as well as the Shapiro–Wilk test were carried out.

## Results

With a dislocation of 0.29 ± 0.15 mm after short-term loading and a dislocation of 0.30 ± 0.12 mm after long-term loading, the tape suture did not display a significant difference regarding horizontal tension (*p* = 0.736). Considering compression, the difference again proved to be non-significant with a dislocation of 0.05 ± 0.02 mm for short term and 0.05 ± 0.01 for long-term loading (*p* = 0.537) (see Table 1).

**Table 1:** Maximum mean displacement (mm)

	Horizontal loading		Vertical loading	
	Compression	Tension	Caudal	Cranial
Short term trial (cycles 1–30)	0.05 ± 0.02	0.29 ± 0.15	0.39 ± 0.11	0.59 ± 0.20
Long term trial (cycles 1001–1030)	0.05 ± 0.01	0.30 ± 0.12	0.32 ± 0.10	0.41 ± 0.10
Significance level (*p*)	*p* = 0.537	*p* = 0.736	*p* = 0.008	*p* = 0.006

For vertical loading, both caudal (*p* = 0.008) and cranial (*p* = 0.006) forces showed a significant difference between short- and long-term loading with a difference of 0.07 mm for caudal and 0.18 mm for cranial loading (see Table 1).

In accordance with the dislocation, the stiffness did not display a significant difference regarding horizontal compression (*p* = 0.517) or tension (*p* = 0.451) but proved to be significant for both cranial and caudal vertical loading (both: *p* < 0.01) (see Table 2). As also seen before regarding dislocation, a slight rise in stiffness can be observed for long term after short-term loading for both cranial and caudal vertical loading (see Table 2).

**Table 2:** Mean stiffness (N/mm)

	Horizontal loading		Vertical loading	
	Compression	Tension	Caudal	Cranial
Short term trial (cycles 1–30)	995.10 ± 245.85	234.30 ± 145.87	138.42 ± 37.18	95.14 ± 40.34
Long term trial (cycles 1001–1030)	1032.12 ± 246.63	195.67 ± 78.67	166.67 ± 38.57	128.96 ± 33.99
Significance level (*p*)	*p* = 0.517	*p* = 0.451	*p* < 0.01	*p* < 0.01

## Discussion

Symphyseal disruptions are most commonly the result of an anteroposterior compression of the pelvic ring and oftentimes require a surgical approach to restore pelvic stability and integrity [2–5]. Currently, the preferred treatment method consists of the implantation of an anterior symphyseal plate osteosynthesis [5], not only bearing major perioperative risks but also resulting in an unnatural iatrogenic arthrodesis of the pubic symphysis as an important biomechanical component of the pelvic ring for physiological force transmission [5, 10].

With regard to the above formulated research questions, we were able to demonstrate that the tape suture construct not only provides sufficient biomechanical stability without compromising the physiological micro mobility of the symphyseal joint but is also able to maintain the given stability without implant loosening or failure when exposed to long-term loading.

Regarding horizontal tension, the tape suture displayed a maximum mean dislocation of 0.29 mm for short term and 0.30 mm for long-term loading. Both values not only reflect the biomechanical stability of the construct but also confirm its avoidance of an iatrogenic arthrodesis as the tape suture, compared to current literature, allows more micro mobility than anterior plating [16]; however, does not exceed the physiological movement of the pubic symphysis of 2 mm [10]. As displayed by the approximately same displacement for short- and long-term loading and the concomitant non-significant difference (*p* = 0.736), the tape suture did not loosen stability after long-term loading.

As expected, horizontal compression displayed the least dislocation with less than 0.1 mm for both loading trials (*p* = 0.537), most probably as a consequence of the symphyseal bone-to-bone contact in that plane and its concomitant self-limitation.

In accordance, the stiffness (N/mm) for horizontal loading found in this study did not differ significantly (*p* > 0.05).

**Fig. 4:**
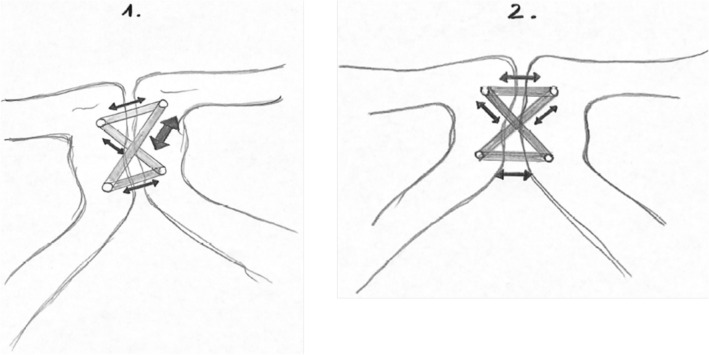
Illustration of the distribution of tension of the tapes before (1) and after (2) initial loading

The biomechanical stability of the tape suture construct is furthermore confirmed by the dislocation found after applying vertical loading (see Table 1), as these values likewise allow more micro mobility than an open reduction and internal fixation using a plate osteosynthesis, yet do not exceed the physiological mobility of 2 mm [10]. Here, both differences were found to be statistically significant with a smaller dislocation for long-term loading (both *p* < 0.01), yet with a minimal difference between the dislocations (caudal: 0.07 mm, cranial: 0.18 mm). This to our minds can be explained by a “settling” of the tapes after initial implantation and exposure to loading. In detail, after initial tape implantation, a difference in tension and concomitantly in force distribution between the tapes can be observed. This, after loading, potentially equalizes, resulting in a more evenly distribution of tension and forces between the four fix points of the tapes and consequently between the tapes themselves (see Fig. 4) as reflected by the slight, yet still significant, biomechanical decrease in dislocation and the increase in stiffness for long-term loading found in this study (*p* < 0.01).

Overall, these results are consistent with the ones of the preliminary study that lay the foundation for this experiment in which the authors were able to demonstrate the feasibility of the tape suture technique in a synthetic pelvic model [16], which’s transferability to an in vitro setting can be confirmed in this study.

Further supporting our idea of a minimally invasive treatment approach with a flexible osteosynthesis technique for symphyseal disruptions, Kiskaddon et al. found comparable biomechanical properties of a suture button fixation technique compared to plate osteosynthesis [21]. However, this approach involves preparation and access to the posterior pubic symphysis and concomitantly potentially endangers the urinary bladder [21], while the tape suture construct evaluated in this study leaves the posterior symphyseal surface unaffected.

Besides, Jordan et al. investigated the implantation of a trans-obturator fixation for symphyseal disruption and reported this cable fixation as a promising alternative to anterior plating based on comparable biomechanical properties [11]. Yet, this technique requires a more extensive surgical approach compared to the tension bands tested in this study and again involves a posterior looping of the cables around the pubic symphysis including the obturator foramen [11], posing a risk for the obturator vessels and nerves which can be avoided by the anterior mounting of the tape suture. Furthermore, the cable fixation as described by the authors poses the challenge of a vertical dislocation due to the exposure to shear forces [11]. This problem can be avoided by the use of anchor systems, ensuring sufficient stability in all planes as demonstrated by our results.

A case report investigating the use of 4.75 mm SwiveLock Anchors^®^ loaded with a FiberTape^®^ in a clinical setting for the treatment of symphyseal instability, yet through a laparoscopic approach, was performed by Arner et al. with satisfying clinical outcome [22]. However, to our minds, the laparoscopic procedure does not necessarily provide any added benefit compared to the anterior minimally invasive approach underlying this study, as not only the presence of a general surgeon is required for sufficient surgical performance, but exact placement of the anchors with sufficient tension is aggravated and the use of fluoroscopy is still not avoided.

Considering the second research question of this study, none of the specimens experienced implant loosening or failure after exposure to long-term loading. While long-term loading and concomitant cutting of the cables into the bone with consecutive impairment of stability has been found to be a challenging complication in previous investigated cerclage and wiring techniques [11, 20], the here implanted anchor systems enforce long-term biomechanical stability due to their entire placement in the pubic bone and their function as a connection link between the tape and the pubic bone.

Another advantage of this flexible osteosynthesis technique lays in the possibility of avoiding revision surgery for implant removal, as currently performed with anterior plating after 6 months [19]. Also, based on the availability of absorbable anchors, the topic of implant removal could prospectively further loose its impact. However, further studies are needed to investigate this question.

In this study, the use of cadaver models enabled in vitro testing and allowed an optimum approach to physiological conditions. Besides, only anterior pelvic rings were used to fully concentrate on the impact of this novel technique on the pubic symphysis, avoiding confounding variables potentially caused by individual force vectors running through the posterior pelvic ring. In detail, the pelvic ring as a complex construct transmits the force from the vertebral spine to the lower extremities, with the anterior pelvic ring contributing approximately 30%; however, still displaying a wide range of individuality [8, 23]. This makes it difficult to evaluate the sole impact of the novel osteosynthesis technique on the pubic symphysis. Therefore, the authors decided on the use of isolated anterior pelvic rings; however, studies investigating the effect of tape sutures on the entire pelvic ring, especially on the sacroiliacal joints, should follow.

Another potential limitation to this study is that there was no direct comparison of the tape suture construct with anterior symphyseal plating. However, the potentially added value is questionable, as there are various studies investigating the biomechanical properties of symphyseal plating, resulting in the widely accepted conclusion of anterior plating being a rigid osteosynthesis, ultimately almost entirely limiting the physiological mobility of the symphyseal joint [11, 16]. Also, the implantation of any other osteosynthesis technique could impair the structure and quality of the cadaver models—a risk the authors were not willing to take for ethical reasons, especially as data for anterior plating, as mentioned above, already exists.

The loading forces applied in this study are based on prior investigations by Meißner et al. and Walheim et al. and have previously been successfully implemented on synthetic pelvic models [10, 16, 20]. As an isolated pubic symphysis without the posterior pelvic ring is not be able to bear physiological loading, symphyseal rupture may occur independently from any applied osteosynthesis [16, 20], whereas the suggested forces of + 75 N and − 75 N for the testing of isolated symphyses that were adopted in this study enable reasonable comparison of the corresponding data.

## Conclusion

In this study, we were able to demonstrate that the tape suture construct provides sufficient biomechanical stability for the treatment of symphyseal instabilities and disruptions in a cadaver model while maintaining micro mobility of the pubic symphysis, ultimately avoiding an unnatural iatrogenic arthrodesis. Furthermore, the minimally invasive tape suture construct is able to resist long-term loading without implant loosening or failure.

## Data Availability

All data are available upon request.
